# Targeting Src homology phosphatase 2 ameliorates mouse diabetic nephropathy by attenuating ERK/NF-κB pathway-mediated renal inflammation

**DOI:** 10.1186/s12964-023-01394-9

**Published:** 2023-12-18

**Authors:** Che Yu, Zhuo Li, Cuili Nie, Lei Chang, Tao Jiang

**Affiliations:** 1 Department of Nephrology, Provincial Hospital Affiliated to Shandong First Medical University, Jinan, Shandong China; 2 Postdoctoral Mobile Station of Shandong University, Jinan, Shandong China; 3 Medical Integration and Practice Center, Cheeloo College of Medicine, Shandong University, Jinan, Shandong China; 4 Division of Pediatrics Neurology, Provincial Hospital Affiliated to Shandong First Medical University, Jinan, Shandong China; 5 Department of Anesthesiology, Shandong Cancer Hospital and Institute, Shandong First Medical University and Shandong Academy of Medical Sciences, 440 Jiyan Road, Huaiyin District, Jinan, 250117 Shandong China

**Keywords:** Diabetic nephropathy, Src homology phosphatase 2, ERK/NF-κB pathway, Renal inflammation

## Abstract

Renal inflammation is a pivotal mechanism underlying the pathophysiology of diabetic nephropathy (DN). The Src homology phosphatase 2 (SHP2) has been demonstrated to be linked to diabetes-induced inflammation, yet its roles and explicit molecular mechanisms in DN remain unexplored. Here, we report that SHP2 activity is upregulated in both DN patients and db/db mice. In addition, pharmacological inhibition of SHP2 with its specific inhibitor PHPS1 alleviates DN in db/db mice and attenuates renal inflammation. In vitro, PHPS1 administration prevents inflammatory responses in HK-2 cells stimulated by high glucose (HG). Mechanistically, PHPS1 represses HG-induced activation of the proinflammatory ERK/NF-κB signaling pathway, and these inhibitory effects are blocked in the presence of an ERK specific inhibitor, hence demonstrating that PHPS1 suppresses ERK/NF-κB pathway-mediated inflammation. Moreover, PHPS1 retards ERK/NF-κB pathway activation in db/db mice, and histologically, SHP2 activity is positively correlated with ERK/NF-κB activation in DN patients. Taken together, these findings identify SHP2 as a potential therapeutic target and show that its pharmacological inhibition might be a promising strategy to mitigate DN.

Video Abstract

**Supplementary Information:**

The online version contains supplementary material available at 10.1186/s12964-023-01394-9.

## Introduction

Diabetic nephropathy (DN) is the leading cause for the end-stage renal disease (ESRD), a common complication of diabetes mellitus (DM) [1]. The pathophysiological mechanisms of DN and induced kidney damage are poorly understood, and effective molecular targets to intervene disease progression are still lacking [2]. Despite current therapies for controlling hypertension, glycemic load and hyperlipidemia, a majority of DN patients progress to the stage of ESRD [3]. To date, the pathogenesis of DN has been closely associated with four causalities, including metabolic, hemodynamic, growth, and proinflammatory factors [4]. Increasing evidence has indicated that renal inflammation plays a central role in DN pathology via regulation of proinflammatory signaling pathways [5, 6]. Therefore, elucidating the molecular mechanisms involved in inflammation-mediated DN progression and kidney injury may identify novel targets for developing preventive or curative therapies for DN.

The Src-homology domain-2-containing protein tyrosine phosphatase 2 (SHP2) is a cytosolic protein tyrosine phosphatase (PTP) expressed in most mammalian tissues and cells [7]. SHP2 is involved in the regulation of growth factor and cytokine signaling pathways, and its mutations account for genetic diseases in the cardiovascular system [8]. Moreover, studies have related SHP2 biological functions to inflammatory and diabetic conditions. For example, SHP2 mediates endothelial inflammation induced by chronic insulin through limiting NO production [9]. In addition, methylglyoxal accumulation induced by hyperglycaemia promotes the resistance of diabetic monocytes to VEGF through SHP2 activation [10]. Further, recent studies have reported that in diabetic patients, SHP2 activates aberrant activation of primary human monocytes [11], and repression of its activity impedes monocyte activation [12]. Despite these activities involved in diabetes-induced inflammation, less is known about whether SHP2 exerts an influence on DN progression and renal injury in diabetic patients and animal models.

In the present study, we took advantage of histologic examinations of human samples, a diabetic mouse model, as well as in vitro cellular studies, and provided some lines of evidence demonstrating that the SHP2 activity was augmented in human DN and db/db mice, as indicated by its elevated phosphorylated level, which promoted renal inflammation through downstream activation of the proinflammatory ERK/NF-κB pathway. Most importantly, we also observed that in db/db mice, renal injury was alleviated by a pharmacological inhibition of SHP2 using its specific inhibitor PHPS1. These results support SHP2 to be a promising therapeutic target in DN treatment.

## Materials and methods

### Antibodies and reagents

Antibodies and reagents were provided from the following sources: phospho-SHP-2 (Tyr542) rabbit mAb, SHP-2 rabbit mAb, phospho-Erk1/2 (Thr202/Tyr204) rabbit mAb, Erk1/2 rabbit mAb, phospho-NF-κB (Ser536) rabbit mAb and NF-κB mouse mAb were obtained from Cell Signaling Technology; CCL-2 mouse mAb was purchased from Invitrogen; β-actin mouse mAb and F4/80 rat mAb were purchased from abcam; SHP2 specific inhibitor PHPS1 and ERK specific inhibitor PD98059 were purchased from Sigma-Aldrich.

### Human kidney samples

Written informed consent was obtained from each patient prior to the sampling of kidney biopsy, which were obtained from 14 patients with DN. Besides, 9 normal nephrectomy samples were used as controls.

### Animal experiments

C57BLKS/J-leprdb/leprdb (db/db) mice and wild-type littermates (WT) were purchased from Nanjing Junke Bioengineering Co., Ltd. Mice were acclimatized to the feeding center for 2 weeks before use. Mice were classified into 4 groups (*n* = 6) at 8 weeks of age, including groups of WT + PHPS1 and db/db + PHPS1 mice injected i.p. with PHPS1 (8 mg/kg body weight) and groups of WT + Ctrl and db/db + Ctrl mice injected i.p. with equal volume of vehicle. Mice were treated every day for 12 weeks. The blood glucose and body weight were measured every two weeks. A metabolic cage was used to collect the twenty-four-hour urine every 2 weeks. Urinary albumin and creatinine levels were determined by enzyme-linked immunosorbent assay (ELISA) kits (abcam). At 20 weeks of age, mice were sacrificed and blood and kidney tissue samples were harvested, which were stored for further usage. Serum creatinine levels in the blood samples were measured through QuantiChrom Aassay Kit (BioAssay Systems). Kidney samples were fixed in paraformaldehyde and embedded within paraffin or frozen in OCT. Tissue Sections with 4-μm thickness were cut. Hematoxylin-eosin, Masson trichrome and periodic acid-Schiff staining was performed to assess renal injury. The intensity of injury was evaluated semiquantitatively by two independent researchers with a blinded manner using a scoring system described in previous studies [13, 14]. The positive immunostaining on kidney sections was semiqualified by Image-Pro Plus Software (Media Cybernetics), and the value was expressed as integrated optical density.

### Cell culture

Human proximal tubular HK-2 cells were purchased from ATCC and cultured at 37 °C in DMEM/F12 (Sigma-Aldrich) medium supplemented with 10% fetal bovine serum (Gibco), 1% penicillin/streptomycin in an atmosphere (5% CO_2_, 95% air). Prior to treatment, HK-2 cells were cultured in serum-free medium for 12 h for starvation, followed by stimulation with 30 mmol/L of high glucose (Sigma-Aldrich) for further 24 h. The control group was cultured with 5.5 mmol/L glucose. HK-2 cells were pretreated with 5 μM PHPS1 alone or in combination with 50 μM PD98059 2 h before glucose stimulation.

### qRT-PCR analysis

Total RNA from kidney tissues and HK-2 cells was isolated by TRIzol method (Invitrogen). Quantitative reverse transcription and PCR (qRT-PCR) analysis was conducted by using an M-MLV Platinum RT-qPCR Kit (Invitrogen) and Eppendorf Real plex 4 instrument (Eppendorf). Specific primers for SHP2, TNF-α, IL-6, CCL-2 and β-actin were purchased from Sangon Biotech. Relative fold change of gene expression was calculated by 2^-ΔΔCT^ method and normalized to that of β-actin.

### Immunoblotting

The concentration of proteins of tissue and cell lysates was determined by Bradford method (Bio-Rad), and then separated by 10% sodium dodecyl sulfate-polyacrylamide gel electrophoresis (SDS-PAGE). Proteins were then transferred to polyvinylidene fluoride membranes (Bio-Rad), which were incubated at room temperature for 1 h in a blocking buffer (5% non-fat milk diluted in Tris-buffered saline containing 0.05% Tween 20). Membranes were probed with specific primary antibodies overnight at 4 °C, and with secondary antibodies conjugated with horseradish peroxidase for another 1 h at room temperature. Immunoreactive bands on the membranes were visualized by enhanced chemiluminescence reagent (Bio-Rad).

### Statistics

All data are expressed as the mean ± SD. Statistical significance was calculated using Student’s test or one-way ANOVA test followed by a Holm-Sidak post-test, as appropriate. All the statistical analyses was conducted with GraphPad Prism 6.0 software (San Diego, CA, USA). A *p* value less than 0.05 was considered statistically significant.

## Results

### SHP2 activity is enhanced in DN patients

**Fig. 1:**
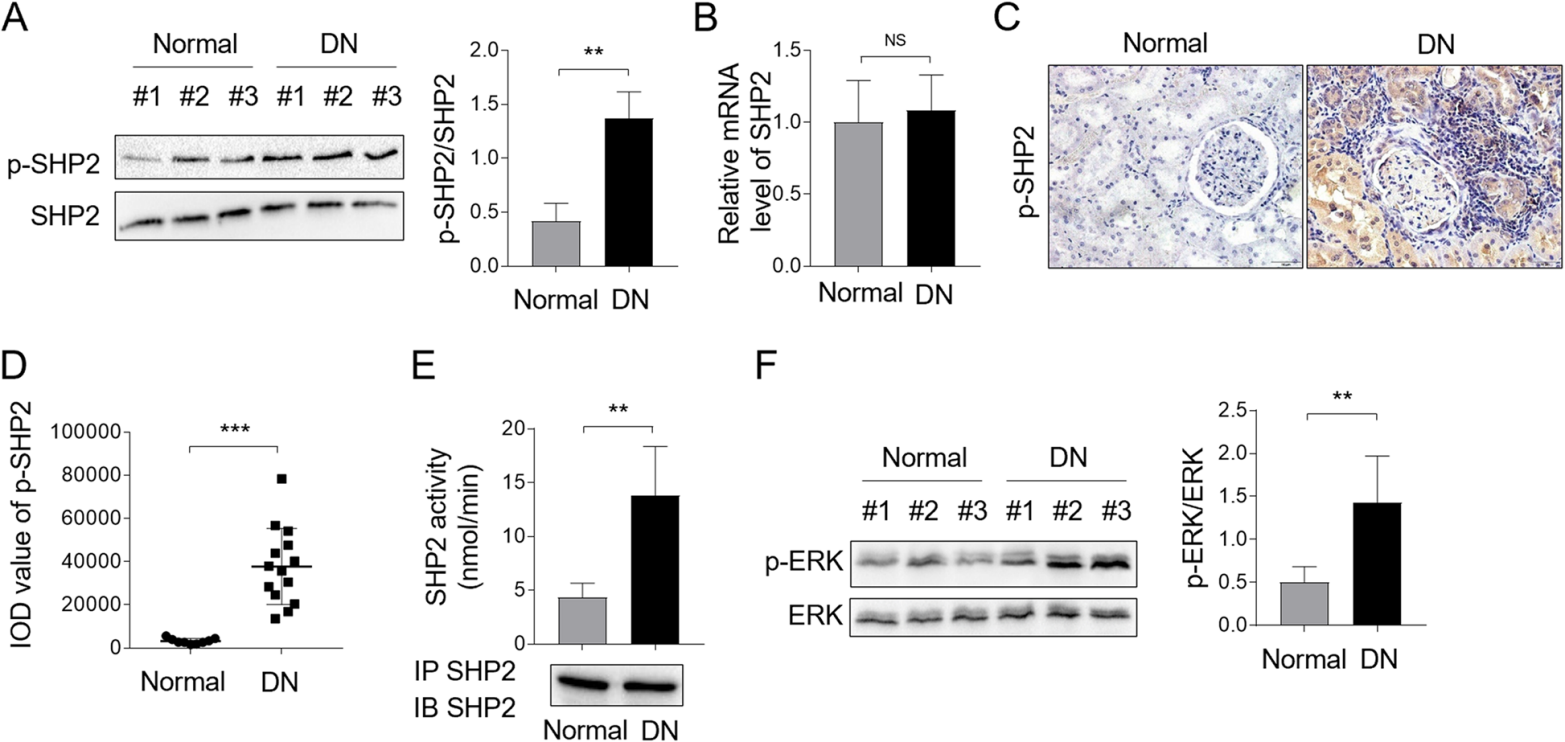
SHP2 is activated in DN patients. **A** levels of p-SHP2 and basal SHP2 protein expression in DN kidney biopsies were measured by Western blot analysis. Normal kidey biopsies were used as control samples. The intensity of potein bands was quntified by ImageJ and the ratio of p-SHP2/SHP2 was shown right. Student’s test, **, *p* < 0.01 (*n* = 6). **B** The mRNA level of SHP2 in DN and normal groups was checked by qRT-PCR analysis. The result relative to the normal group was depicted. Student’s test, NS, not significant (*n* = 6). **C**, **D** Immunostaining of p-SHP2 on normal kidney sections (*n* = 9) and kidney biopsy samples of DN patients (*n* = 14) was visualized by IHC experiment (**C**). The integrated optical density (IOD) of p-SHP2 expression in (**C**) was shown (**D**). One-way ANOVA test followed by a Holm-Sidak post-test, ***, *p* < 0.001. **E** SHP2 immune complex PTP assay was performed using pNPP as a substrate on human DN samples (n = 9) and normal controls (*n* = 6). Immunoblotting control for immunoprecipitated SHP2 was shown below. One-way ANOVA test followed by a Holm-Sidak post-test, **, *p* < 0.01. **F** Levels of p-ERK and basal ERK protein expression in DN kidney biopsies were measured by Western blot analysis. Normal kidey biopsies were used as control samples. The intensity of potein bands was quntified by ImageJ and the ratio of p-ERK/ERK was shown right. Student’s test, **, *p* < 0.01 (*n* = 6)

To evaluate whether SHP2 is involved in DN pathogenesis, we conducted Western blot analysis to determine its activity (represented as the level of phosphorylated SHP2, p-SHP2) in kidney biopsy samples of DN patients, and normal kidney biopsy samples were used as controls. The result showed that the ratio of p-SHP2/SHP2 was markedly elevated in DN samples compared with that in normal controls (Fig. 1A), suggesting that SHP2 is activated in DN patients. Of note, we did not observe upregulation of basal expression level of SHP2 in DN patients, as shown by qRT-PCR analysis (Fig. 1B). To confirm these data, we performed immunostaining of p-SHP2 on DN samples through immunohistochemistry tecnique (IHC). As shown in Fig. 1C-D, p-SHP2 staining was notably enhanced in DN samples in comparison with normal kidney biopsies, showing predominantly cytosolic location. Moreover, we took advantage of an immune-complex phosphatase assay to measure SHP2 activity in kidney biopsy samples with DN, and found that SHP2 activity was indeed increased significanlty (nearly 3.2-fold) in DN as compared with normal ones (Fig. 1E), further proving that SHP2 activity is strengthened in DN. SHP2 is well-established to ignite downstream activation of ERK mitogen-activated protein kinase in multiple pathways [15–17]. To confirm the activation of SHP2, we further checked the status of ERK in DN samples with an antibody probing ERK phosphrylation. As expected, consistent with SHP2 activation, the phosphrylated level of ERK (p-ERK) was also increased in DN in contrast to the normal group (Fig. 1F), suggesting an acivated SHP2/ERK pathway in DN condition. Collectively, these results indicate that SHP2 is activated in DN patients, implying a potential role of SHP2 involved in DN pathogenesis.

### SHP2 is activated in diabetic mice

**Fig. 2:**
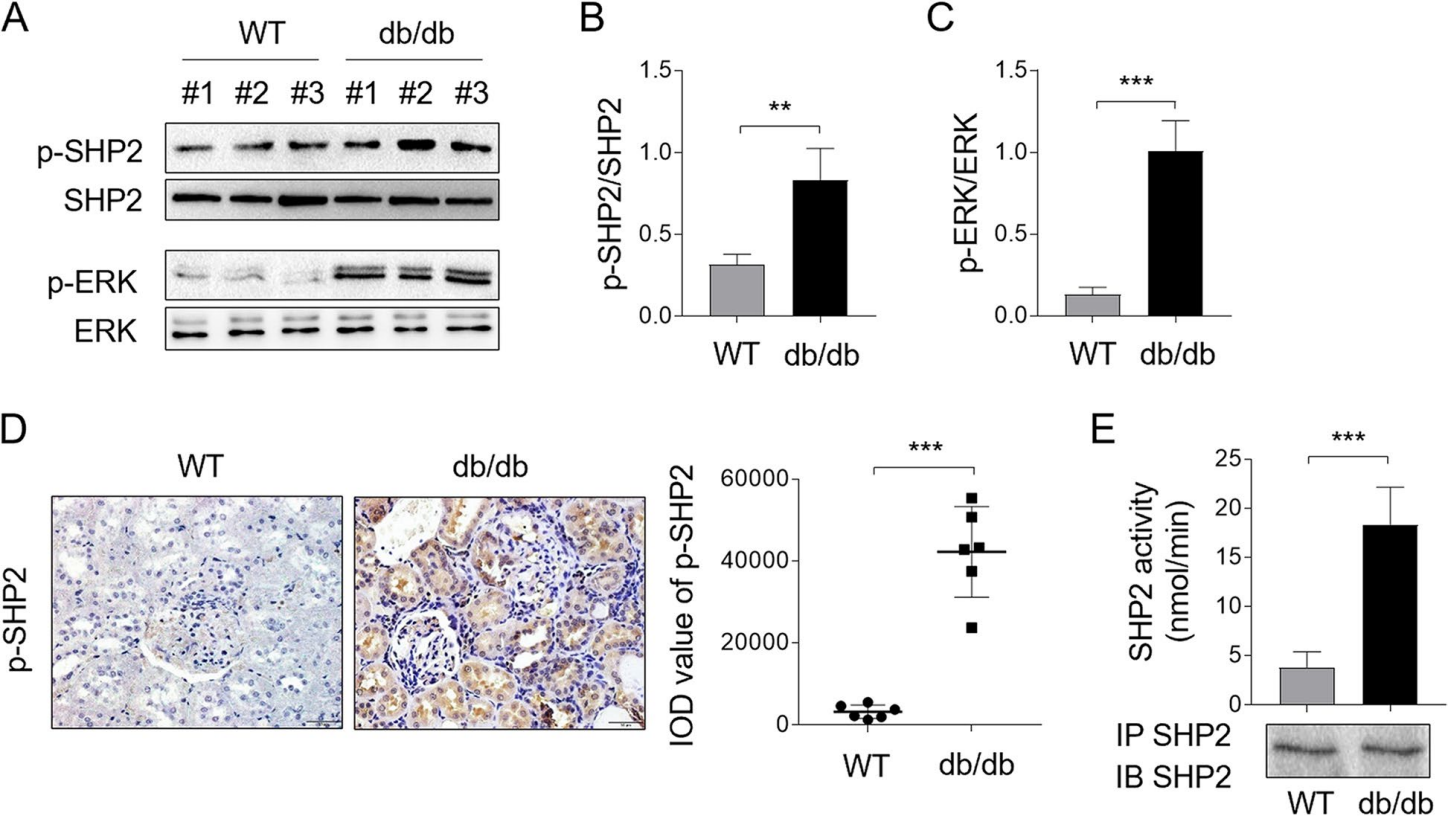
SHP2 is activated in db/db mice. **A** Levels of p-SHP2, p-ERK and their basal protein expression in kidney biopsies of db/db mice were measured by Western blot analysis. Normal kidey biopsies of WT mice were used as control samples. **B**, **C** The intensity of potein bands (**A**) was quntified by ImageJ and the ratio of p-SHP2/SHP2 (**B**) and p-ERK/ERK (**C**) was shown right. Student’s test, **, *p* < 0.01; ***, *p* < 0.001 (*n* = 6). **D** Immunostaining of p-SHP2 on mouse normal kidney sections (*n* = 6) and kidney biopsy samples of db/db mice (*n* = 6) was detected by IHC experiment. The integrated optical density (IOD) of p-SHP2 expression was shown right. Student’s test, ***, *p* < 0.001. **E** SHP2 immune complex PTP assay was performed using pNPP as a substrate on normal kidney samples (*n* = 6) from WT mice and DN kidney samples from db/db mice (*n* = 6). Immunoblotting control for immunoprecipitated SHP2 was shown below. Student’s test, ***, *p* < 0.001

We next used db/db mice, a murine model that recapitulates human DN [18], to investigate a potential connection of SHP2 to animal DN pathology. The blood glucose levels and body weight of mice were monitored, demonstrating typical changes in mice with DN compared to wild-type mice (Supplementary Fig. 1). Likewise, Western blot analysis showed that both the phosphorylation levels of SHP2 and downstream ERK were increased significantly in db/db mice, in contrast to wild-type mice (WT) (Fig. 2A), and the ratio of p-SHP2/SHP2 (Fig. 2B) and p-ERK/ERK (Fig. 2C) was also prominently increased in db/db mice. In agreement with these, IHC staining on kidney samples of mice also showed an overt upregulation of p-SHP2 in db/db mice (Fig. 2D). Consistent with these data, the immune-complex phosphatase assay uncovered that the activity of SHP2 in kidney samples from db/db mice was drastically augmented compared with that of WT mice (Fig. 2E). Hence, similar to those observations of human DN, the activity of SHP2 is also amplified in mouse DN model, together demonstrating an association of activated SHP2 with DN pathology.

### SHP2 inhibition with PHPS1 alleviates renal injury in db/db mice

**Fig. 3:**
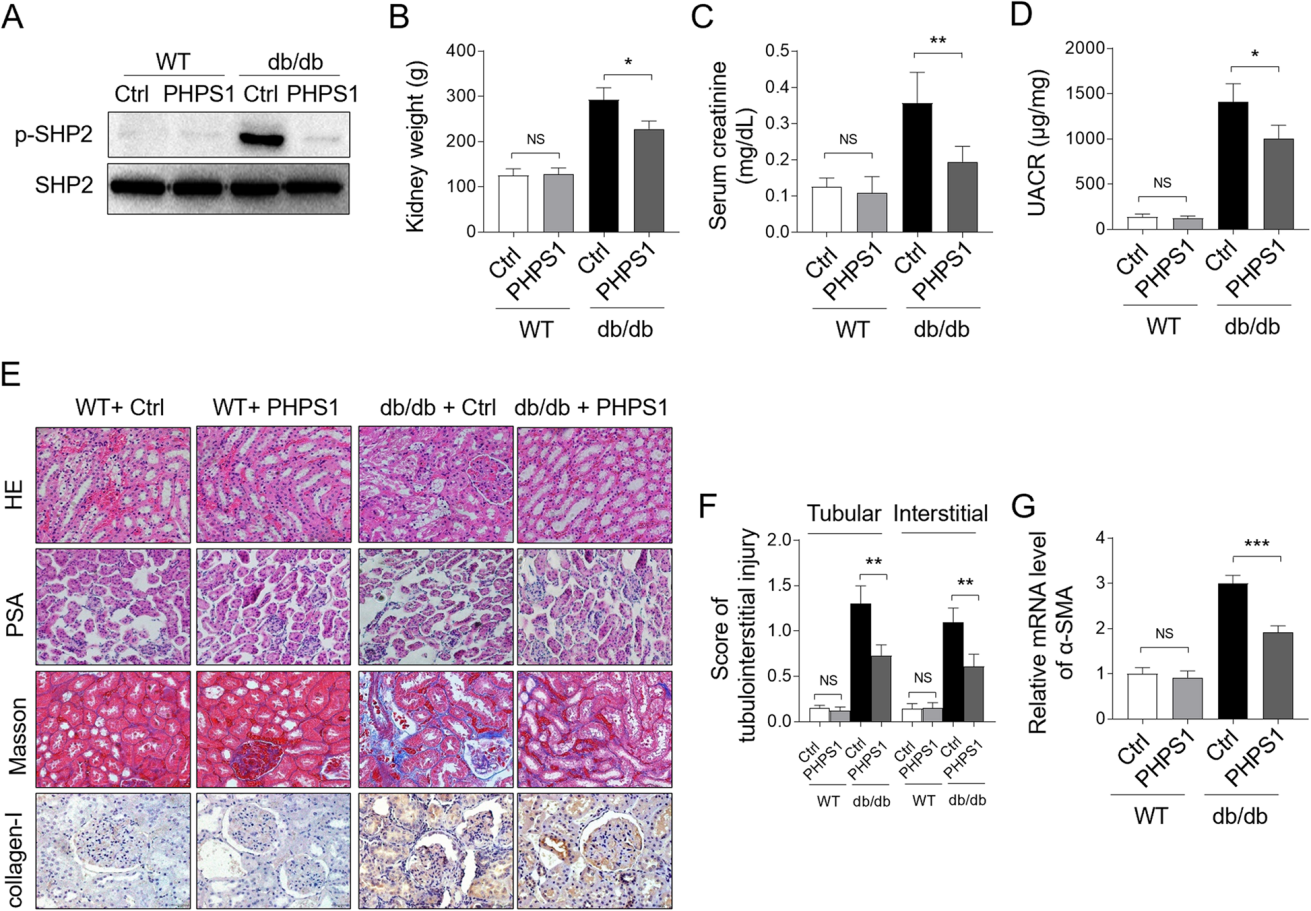
PHPS1 alleviates renal injury in db/db mice. **A** WT and db/db mice were adminstrated i.p. with 8 mg/kg/d PHPS1 for 12 weeks, starting at 8 weeks to 20 weeks of age. Injecton of equal volume of vehicle was used as control (Ctrl). Each group included 6 mice. The levels of p-SHP2 and basal SHP2 protein were determined by Western blot analysis. **B-D** Mice were treated as in (**A**). Mouse kidneys in each group were harvested and weighted (**B**). The urinary albumin and creatinine levels were measured using enzyme-linked immunosorbent assay (ELISA), and the serum creatinine level (**C**) and urine albumin-to-creatinine ratio (UACR) (**D**) were presented. Student’s test, *, *p* < 0.05; **, *p* < 0.01; NS, not significant (*n* = 6). **E** Histologic examinations of renal sections from each group were performed via hematoxylin and eosin (HE) staining and periodic acid-Schiff (PAS) staining. Masson trichrome staining and IHC staining of collagen-I were employed to measure collagen fiber deposition. **F** The tubulointerstitial injury in (E) was semiquantified and the score in each group was depicted. Student’s test, **, *p* < 0.01; NS, not significant (*n* = 6). **G** The mRNA level of α-SMA in renal tissue was detected by qRT-PCR analysis. Student’s test, ***, *p* < 0.001; NS, not significant (*n* = 6)

To explore a potential protective effect of counteracting SHP2 activation on renal injury in db/db mice, we utilized a pharmacological inhibition strategy in which PHPS1, a specific inhibitor of SHP2 [19], was administered by i.p. injection (8 mg/kg/day) to db/db mice for 12 weeks. Western blot analysis of the whole lysates of kidney tissue demosntrated an effective inhibition of SHP2 activation in db/db mice in the presence of PHPS1 administration, as shown by drastic decrease level of p-SHP2 (Fig. 3A). Additionally, PHPS1 treeatment resulted in significant loss of kidney weight in db/db mice, without causing evident change in WT mice (Fig. 3B). Importantly, the levels of serum creatinine (Fig. 3C) and urine albumin-to-creatinine ratio (UACR) (Fig. 3D) in db/db mice were sharply decreased upon PHPS1 treeatment, while no obvious changes were observed in WT mice. Moreover, histological analyses of renal sections through PAS staining and hematoxylin and eosin (HE) staining displayed glomerulosclerosis, glomerular vascular tufts, atypical tubular epithelia and interstitial expansion in diabetic db/db mice, while these stractual changes were reduced by PHPS1 treeatment (Fig. 3E-F). Furthermore, increased fibrosis and collagen deposition in the interstitial compartment of kidneys from diabetic db/db mice were stained by Masson’s trichrome, which was further verified by type I collagen (collagen-I) staining, indicating a significant increase of interstitial fibrosis and tubular atrophy (IFTA) in db/db mice, while these renal tubulointerstitial injuries were alleviated by PHPS1 treeatment (Fig. 3E-F). Consistent with these histologic results, the elevated mRNA expression level of the profibrotic molecule alpha-smooth muscle actin (α-SMA) in the kidneys of db/db mice was distinctly prevented upon PHPS1 administration (Fig. 3G). Altogether, these observations suggest that SHP2 inhibition with PHPS1 is able to mitigate renal injury in db/db mice.

### PHPS1 administration attenuates renal inflammation in db/db mice

**Fig. 4:**
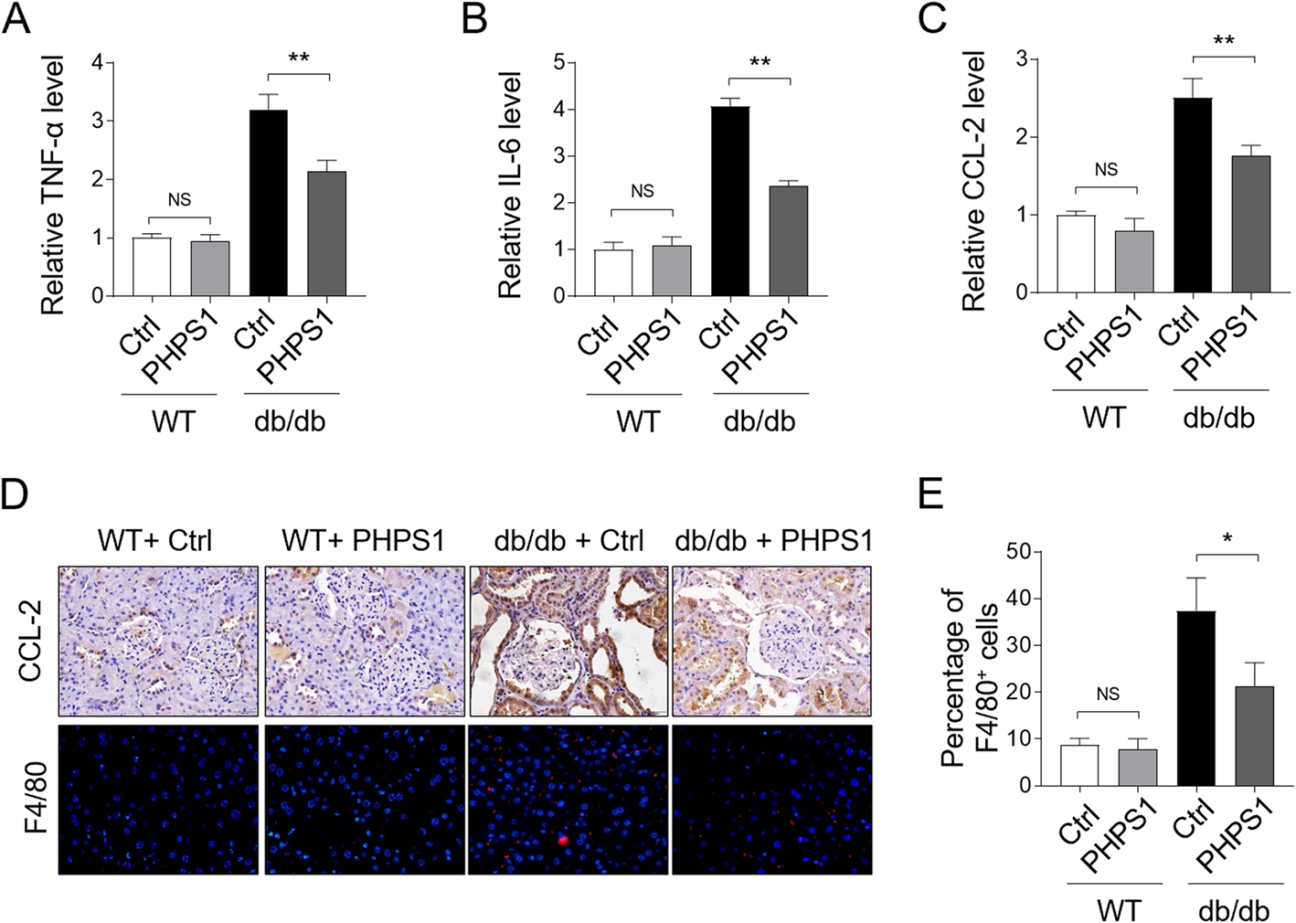
PHPS1 alleviates renal inflammation in db/db mice. **A-C** WT and db/db mice were adminstrated i.p. with 8 mg/kg/d PHPS1 for 12 weeks, starting at 8 weeks to 20 weeks of age. Injecton of equal volume of vehicle was used as control (Ctrl). Each group included 6 mice. The mRNA level of TNF-α (**A**), IL-6 (**B**), and CCL-2 (**C**) in renal tissue of each group mice was quantified by qRT-PCR analysis. Student’s test, **, *p* < 0.01; NS, not significant (*n* = 6). **D** CCL-2 in kidney tissue isolated from each group was stained by IHC experiment (upper). The immunofluorescent detection of F4/80 (red dots) was used to evaluate renal macrophage infiltration (lower). **E** The number of F4/80^+^ infiltrated macrophages as shown in (**D**) was quantified and the percentage of these cells in each group was presented. Student’s test, *, *p* < 0.05; NS, not significant (*n* = 6)

It has been documented that SHP2 is able to regulate some inflammatory diseases [20]. In order to investigate whether the protective effect of SHP2 inhibition against renal injury in DN is attributed to its anti-inflammatory activities, we checked the levels of some important proinflammatory factors in the kidney tissue. qRT-PCR analysis manifested that the levels of the tumor necrosis factor-a (TNF-α) (Fig. 4A), interleukin (IL)-6 (Fig. 4B), as well as CC chemokine ligand (CCL)-2 (Fig. 4C) were all induced in db/db mice, whereas SHP2 inhibition with PHPS1 markedly reduced the level of these proinflammatory cytokines. IHC staining of renal sections also displayed that PHPS1 treatment prevented CCL-2 induction in db/db mice (Fig. 4D). In concernt with these findings, the percentage of F4/80^+^ macrophage infiltrated into the renal tissue was remarkably decreased by PHPS1 in the tubulointerstitium of db/db mice (Fig. 4D-E). In sum, these findings indicate that the along with alleviated renal injury, the renal inflammation in db/db mice mediated by proinflammatory factors is also dampened by SHP2 inhibition with PHPS1.

### PHPS1 treatment inhibits high glucose-induced inflammatory responses in vitro

**Fig. 5:**
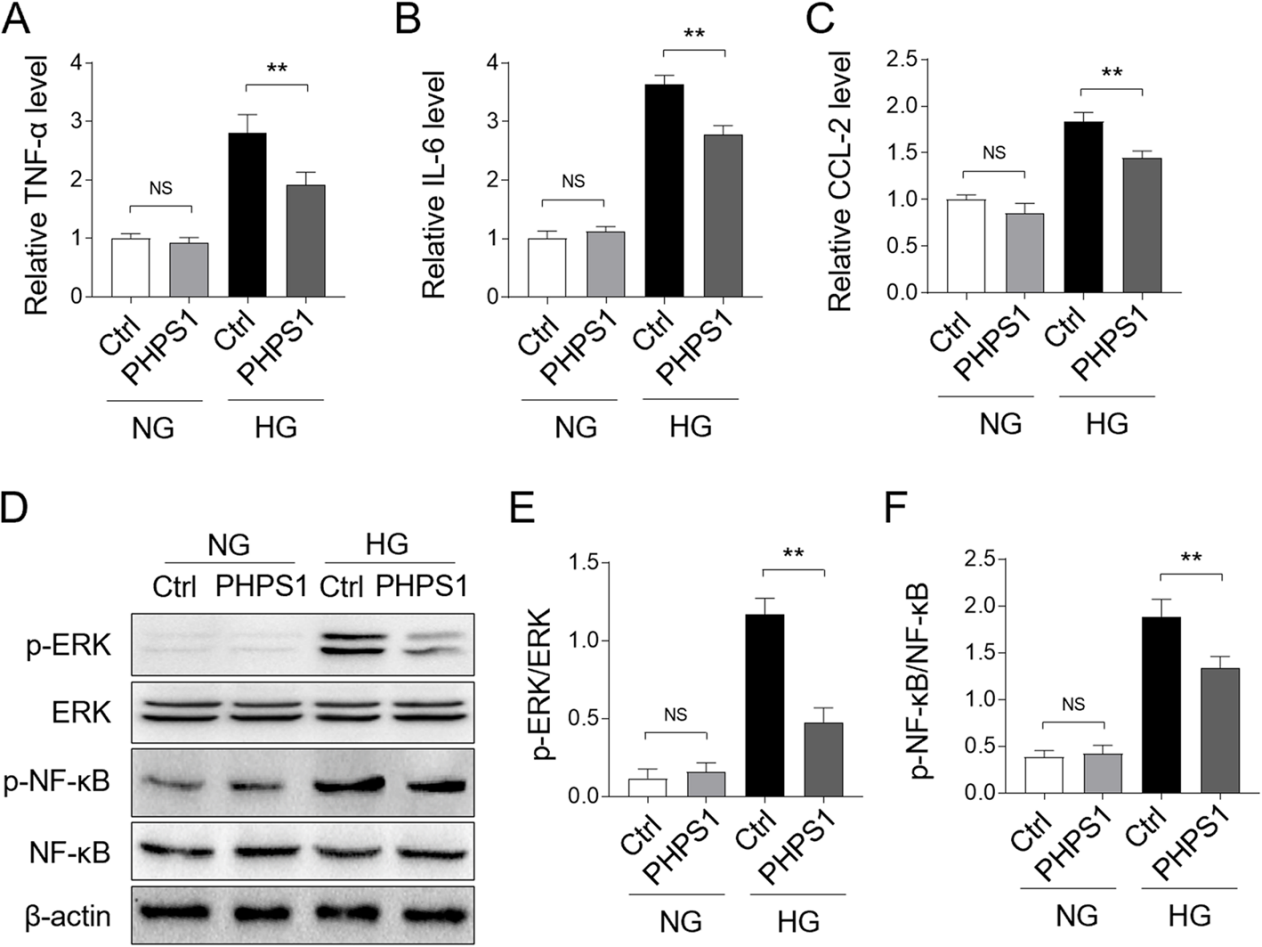
PHPS1 decreases HG-induced inflammatory responses in HK-2 cells in vitro. **A**-**C** HK-2 cells were pretreated with 5 μM PHPS1 before exposure to high glucose (HG, 30 mM) for 24 h. The control group was incubated with normal glucose (NG, 5.5 mM). The mRNA level of TNF-α (**A**), IL-6 (**B**), and CCL-2 (**C**) in each group of HK-2 cells was quantified by qRT-PCR analysis. Student’s test, **, *p* < 0.01; NS, not significant (*n* = 3). **D**-**F** HK-2 cells were treated as in (A-C), the levels of p-ERK, p-NF-κB and their basal protein expression were measured by Western blot analysis. The representative images were shown (**D**). The intensity of potein bands (**D**) was quntified by ImageJ and the ratio of p-ERK/ERK (**E**) and p-NF-κB/ NF-κB (**F**) were depicted. Student’s test, **, *p* < 0.01; NS, not significant (*n* = 3)

We next extended our findings in vitro using HK-2 cells, immortalized proximal tubule epithelial cells establised from normal adult human kidney [21], which were exposed to high glucose (HG, 30 mM) culture condition. We found that pre-treatment with PHPS1 prevented elevated production of proinflammatory cytokines by HG stimulation, including TNF-α (Fig. 5A), IL-6 (Fig. 5B), as well as CCL-2 (Fig. 5B), indicating that HG-induced inflammatory responses were repressed by PHPS1 treatment. In consistence with these findings, the activated proinflammatory pathway ERK/NF-κB by high glucose exposure was hindered by PHPS1 in HK-2 cells, as shown by markedly reduced level of p-ERK and p-NF-κB (Fig. 5D) and the coresponding decreased ratio of p-ERK/ERK (Fig. 5E) and p-NF-κB/NF-κB (Fig. 5F). Taken together, these results unveil that SHP2 inhibitor PHPS1 ameliorates high glucose-induced inflammation and blocks the activation of proinflammatory ERK/NF-κB pathway in HK-2 cells.

### PHPS1 prevents high glucose-induced inflammatory responses through suppressing ERK/NF-κB pathway

**Fig. 6:**
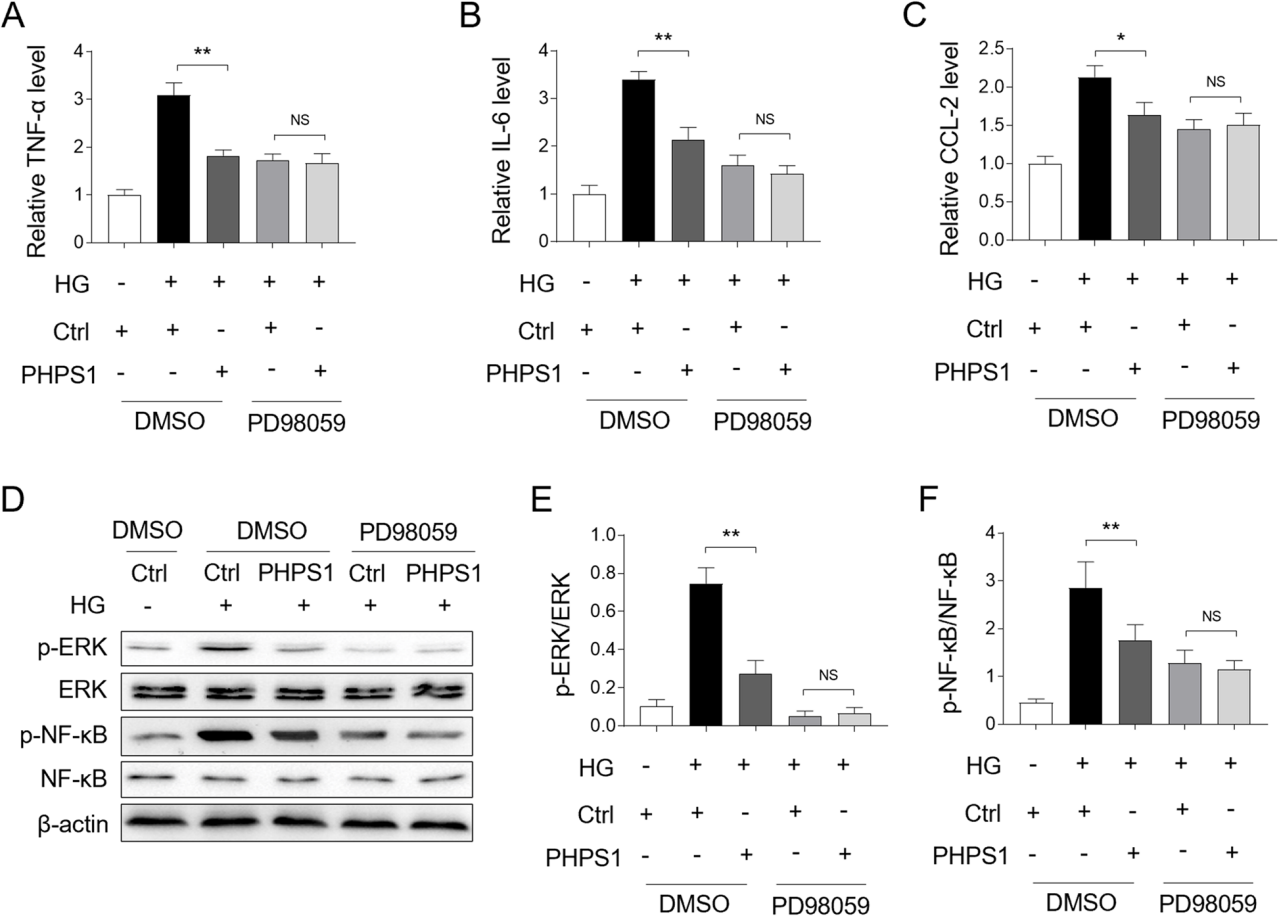
PHPS1 prevents high glucose-induced inflammatory responses through ERK/NF-κB pathway. **A-C** HK-2 cells were pretreated with 5 μM PHPS1 alone or in combination with 50 μM PD98059 before exposure to high glucose (HG, 30 mM) for 24 h. The control group was incubated with normal glucose (NG, 5.5 mM). The mRNA level of TNF-α (**A**), IL-6 (**B**), and CCL-2 (**C**) in each group of HK-2 cells was quantified by qRT-PCR analysis. Student’s test, **, *p* < 0.01; *, *p* < 0.05; NS, not significant (*n* = 3). **D**-**F** HK-2 cells were treated as in (A-C), the levels of p-ERK, p-NF-κB and their basal protein expression were measured by Western blot analysis. The representative images were shown (**D**). The intensity of potein bands (**D**) was quntified by ImageJ and the ratio of p-ERK/ERK (**E**) and p-NF-κB/ NF-κB (**F**) were depicted. Student’s test, **, *p* < 0.01; NS, not significant (*n* = 3)

The activation of ERK/NF-κB pathway is important for mediating inflammation in a variety of settings [22–24]. In light of the above finding that the ERK/NF-κB pathway was inhibited by PHPS1, we wondered whether this pathway accounted for the anti-inflammatory effect of PHPS1 in HK-2 cells. To test this idea, we pretreated HK-2 cells with a specific ERK pathway inhibitor PD98059 to eliminate the influence of this pathway on PHPS1 activity. As shown in Fig. 6A, PHPS1 treatment alone consistently attenuated TNF-α induction in HG- stimulated HK-2 cells, while combined treatment with PD98059 could no longer exert similar anti-inflammatory activity. Likewise, PHPS1 treatment leaded to no significant changes in the induction of IL-6 (Fig. 6B) and CCL-2 (Fig. 6C) in the presence of PD98059. These results describe that PHPS1-prevented inflammatory responses rely on inhibited ERK pathway. This notion was further strengthened by the evidence that PHPS1 failed to restrict the activation of ERK/NF-κB pathway in combination with PD98059 pretreatment, compared with PHPS1 treatment alone, as shown by Western blot analysis (Fig. 6D) and the ratio of p-ERK/ERK (Fig. 6E) and p-NF-κB/NF-κB (Fig. 6F). Hence, the ERK/NF-κB pathway regulates the anti-inflammatory effect of PHPS1 on HK-2 cells exposed to HG.

### PHPS1 limits ERK/NF-κB activation in db/db mice

**Fig. 7:**
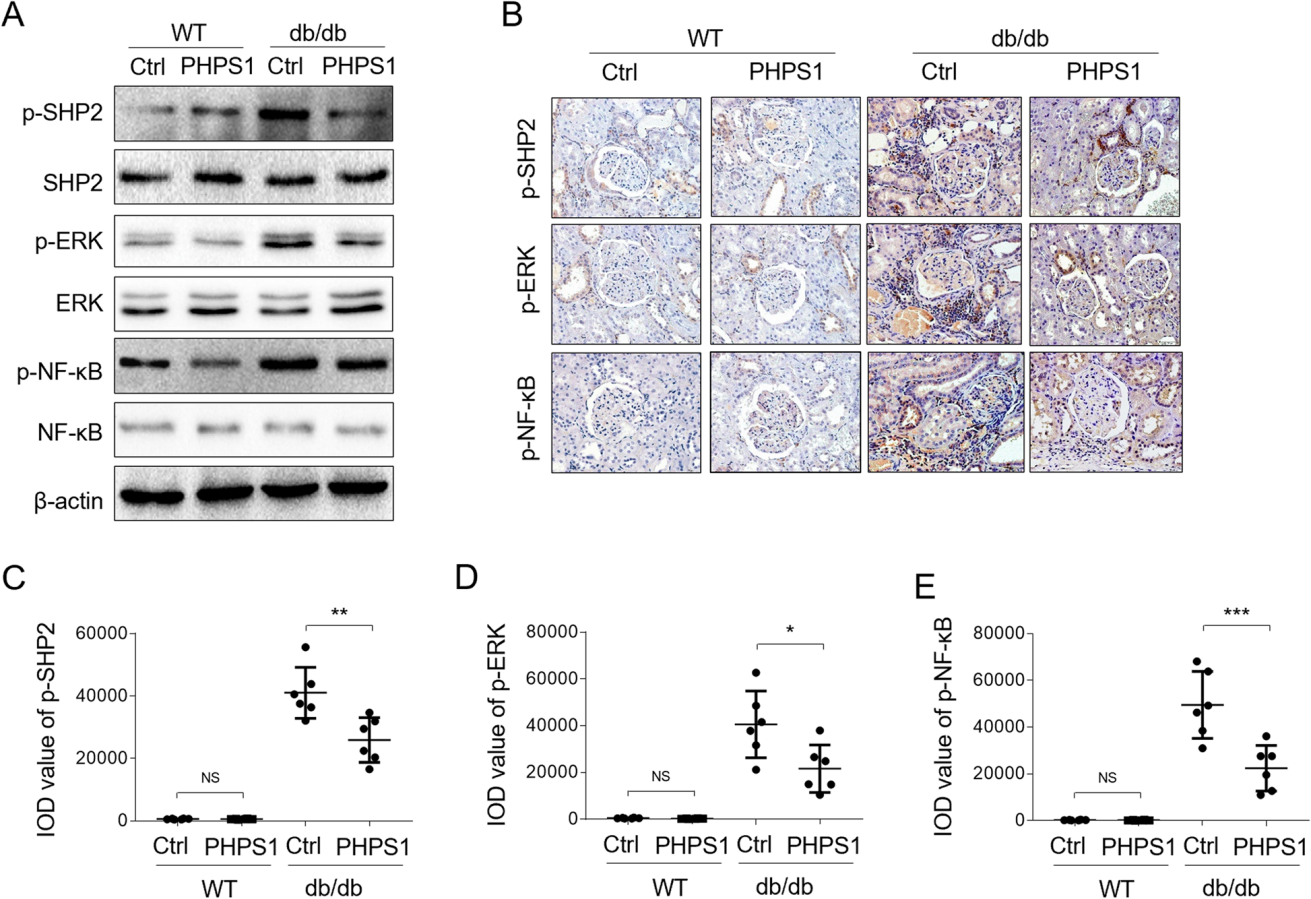
ERK/NF-κB pathway is constrained by PHPS1 in db/db mice. **A** WT and db/db mice were adminstrated i.p. with 8 mg/kg/d PHPS1 for 12 weeks, starting at 8 weeks to 20 weeks of age. Injecton of equal volume of vehicle was used as control (Ctrl). Each group included 6 mice. The levels of p-SHP2, p-ERK, p-NF-κB and their basal proteins were determined by Western blot analysis, and the reprentative images were shown. **B** Immunostaining of p-SHP2, p-ERK, p-NF-κB on mouse normal kidney sections (*n* = 6) and kidney biopsy samples of db/db mice (*n* = 6) was detected by IHC experiment. **C**-**E** The integrated optical density (IOD) of expression of p-SHP2 (**C**), p-ERK (**D**), and p-NF-κB (**E**) was shown. Student’s test, ***, *p* < 0.001; **, *p* < 0.01; *, *p* < 0.05

To establish a causal link between protective activity of PHPS1 against renal injury and ERK/NF-κB pathway modulation, we then examined whether PHPS1 inhibits ERK/NF-κB pathway in vivo. Through checking the phosphrylated proteins via Western blot analysis on kidney tissue samples, we noticed that along with the decreased level of p-SHP2 in db/db mice by PHPS1 administration, the levels of and p-ERK and p-NF-κB were accordingly downregualted (Fig. 7A). To verify this, we further detected the expression of these phosphrylated proteins in kidney sections by IHC assay, and similar tendency was obtained, as indicated by significantly reduced levels of p-SHP2 (Fig. 7B and C), p-ERK (Fig. 7B and D), and p-NF-κB (Fig. 7B and E) in db/db mice administrated with PHPS1. Therefore, PHPS1 also negatively regulates the activation of ERK/NF-κB pathway in db/db mice.

### SHP2 activation correlates positively with ERK/NF-κB activation in DN patients

**Fig. 8:**
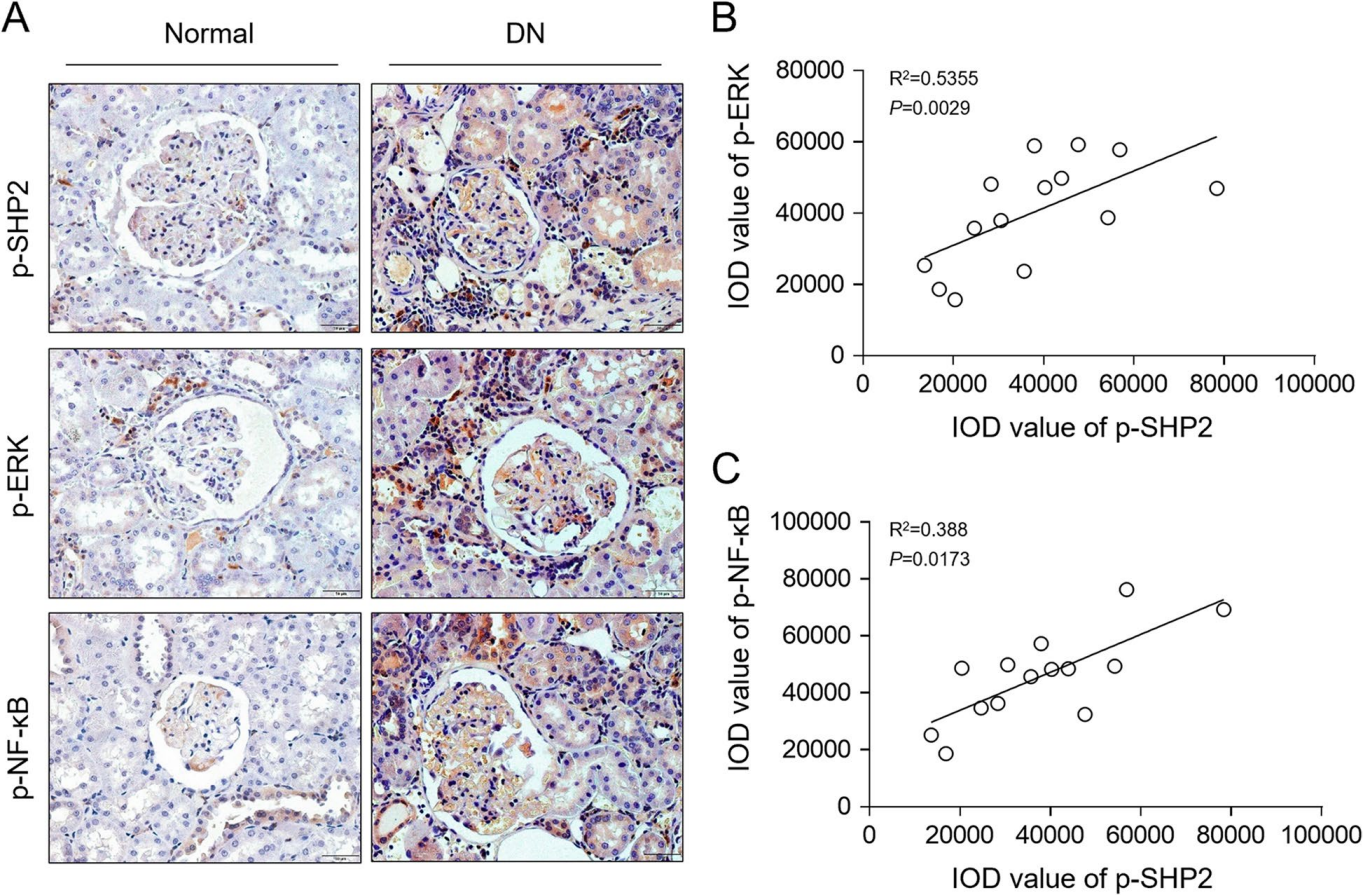
SHP2 activation correlates positively with ERK/NF-κB activation in DN patients. **A** Immunostaining of p-SHP2, p-ERK and p-NF-κB on kidney biopsy samples of DN patients (*n* = 14) was visualized by IHC experiment. **B**, **C** Correlation analysis of integrated optical density (IOD) of p-SHP2 expression with that of p-ERK (**B**) and p-NF-κB (**C**). Pearson’s correlation analysis

To understand the relation of the activation status of SHP2 and ERK/NF-κB pathway in human DN pathology, we performed immunostaining of p-SHP2, p-ERK and p-NF-κB on human DN samples through IHC (Fig. 8A), and further analyzed the correlation between their expression levels. The results illustrated that p-SHP2 level was positively accociated with that of p-ERK (Fig. 8B) and p-NF-κB (Fig. 8C). Moreover, consistent with these, immunohistochemical staining of CD3^+^ (T lymphocytes) and chemokine (C-C motif) ligand 2/monocyte chemoattractant protein-1 (CCL-2/MCP-1) in the kidney showed that their expression was much more pronounced in DN kidney sections than in the normal kidney sections (Supplementary Fig. 2). Based on the above observations that SHP2 inhibition with PHPS1 limited ERK/NF-κB activation and suppresed inflammation in db/db mice and in vitro condition, these results together imply that the activation of SHP2 positively regulates the ERK/NF-κB pathway to induce renal inflammation in DN, whereby promoting disease progression and renal injury.

## Disscussion

**Fig. 9:**
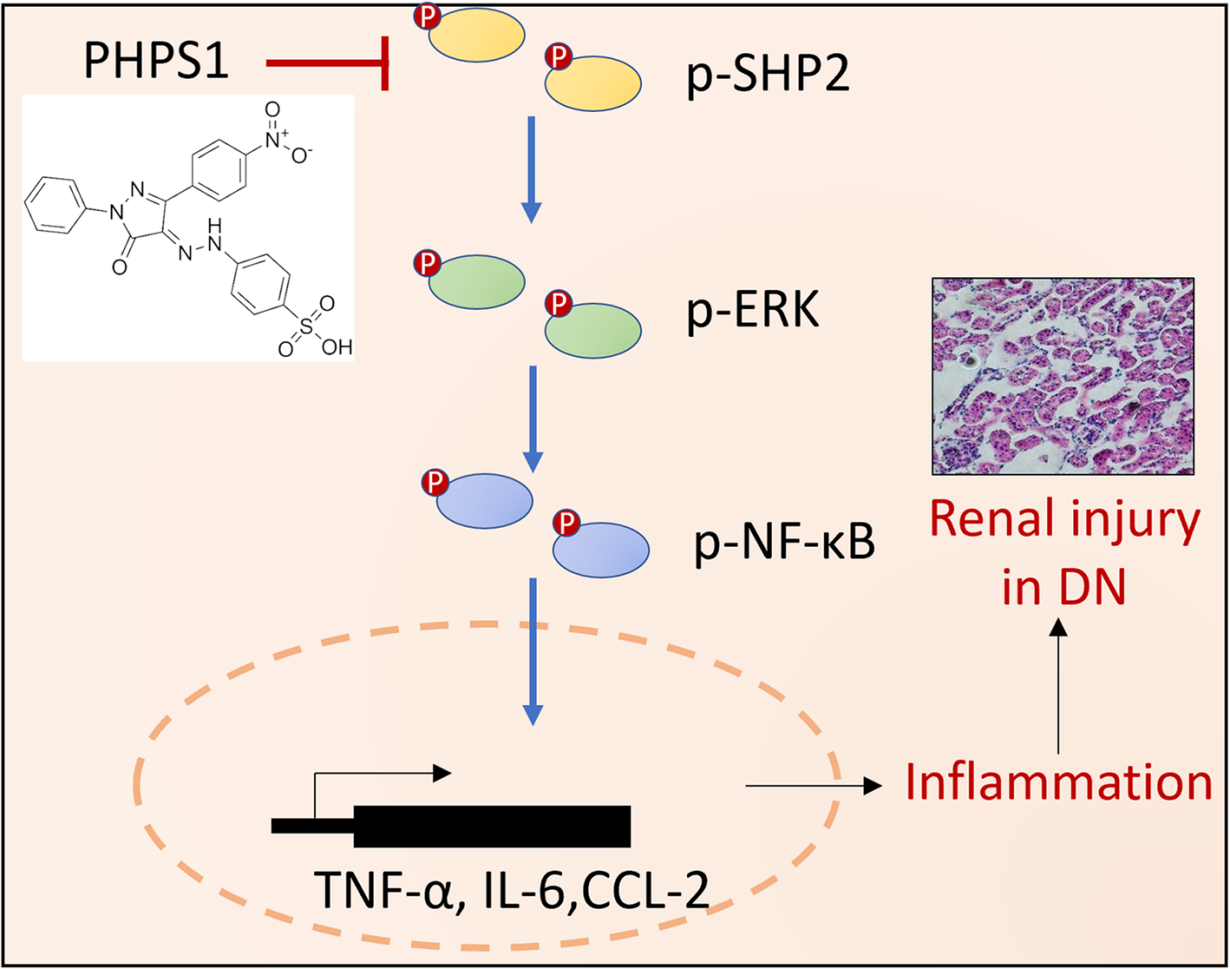
Schematic diagram illustrating the mechanism of action of the therapeutic effect of PHPS1 in DN. PHPS1 inhibition of SHP2 activity ameliorates renal injury in DN by attenuating ERK/NF-κB pathway-mediated inflammation

Increasing studies have demonstrated an important role of inflammation in DN pathogenesis and renal damage, however the involved regulatory factors and underlying mechanisms are no fully unclear. In the current study, we provided some lines of evidence in the first time revealing that the activity of a cytosolic protein tyrosine phosphatase, i.e., SHP2, was enhanced in kidney biopsies of DN patients as well as db/db mice. Through a pharmacological inhibition strategy of SHP2 using PHPS1 administrated in an animal mode of DN, we demonstrated that inhibiting SHP2 activity yielded therapeutic efffects on alleviating renal injury and attenuating renal inflammation in vivo, hence offering SHP2 as a new potential target in DN therapy. To gain molecular insights into the SHP2 regulation of inflammation, we elucidated the signaling pathway through which SHP2 regulates inflammation in HK-2 cells stimulated by high glucose in vitro, and discovered that SHP2 inhibition by PHPS1 prevented high glucose-induced inflammatory responses through suppressing the proinflammatory ERK/NF-κB pathway. More importantly, in accordance with this molecular mechanism, the activation of ERK/NF-κB pathway was similarly suppressed by PHPS1 in db/db mice in vivo, and histologic examinations of human DN sampels further revealed a positive correlation between SHP2 activity and ERK/NF-κB activation. In conclusion, our study reports a therapeutic effect of pharmacological targeting of SHP2 on renal injury in an animal DN model, and also highlights a critical role of SHP2/ERK/NF-κB pathway-induced production of proinflammatory cytokines and -elicited inflammation in promoting renal injury in DN pathology (Fig. 9).

SHP2 is a protein tyrosine phosphatase widely expressed in most tissues and cells and functions to regulate cytokine, integrin, and tyrosine receptor signaling pathways. Its aberration has a causal link to multiple diseases including systemic lupus erythematosus, cancer, and other genetic disorders [25–28]. Studies have also shown that SHP2 can regualte some inflammatory diseases, such as cancer-related inflammation and metabolic diseases [20]. We found that the activity of SHP2 was induced in DN without the change of its expression level, which extents its scope of functions involved in inflammatory and metabolic diseases. However, the upstream regulator(s) activating SHP2 are unclear. SHP2 activation can be regulated by different receptor tyrosine kinases (RTKs), which recruit SHP2 to the plasma membrane for its activation in response to cellular signals, for example the stimulation of cytokines [29]. Within the local pro-inflammatory niche of injured kidney tissue with DN, inflammatory cells infiltrate the site of injury and result in profibrotic cytokine pressure [30], it may thus be plausible that the observed activation of SHP2 in DN is at least partly attributed to stimulation of RTKs by local cytokines. Besides, studies have shown that the protein tyrosine kinases (PTKs) can also regulate SHP2 activity through multiple signaling pathways [31, 32], making the regulation network of SHP2 highly complicated. Further investigations on seeking SHP2 activator(s) in DN scenario are needed.

PHPS1 was identified as an active site-directed small molecule inhibitor of SHP2 in 2008 [19]. Since its discovery, PHPS1 has been demonstrated to show ameliorating effect in multiple diseases in animal models, such as acute kidney injury [33], atherosclerosis [34], cardiac hypertrophy [35], pulmonary arterial hypertension [36], and systemic lupus erythematosus [25]. In our study, PHPS1 treatment va i.p. injection alleviated renal injury and exerted anti-inflammatory activity in db/db mice, hence suggesting it as a promising therapeutic in DN treatment. Together with previous studies mentioned above, these findings support a wide spectrum of PHPS1 utility in the treatment of SHP2-related human diseases.

The ERK/NF-κB pathway is a well-establised proinflammatory pathway responsible for proinflammatory cytokine production and inflammation initiation [22, 37–41]. In addition, two earlier studies have shown that SHP2 phosphorylation is critical for ERK activation in response to growth factors by controlling Csk recruitment [15, 16]. Consistent with these hints, we found that SHP2 inhibition suppressed ERK/NF-κB pathway activation, and further confirmed its important role in limiting proinflammatory responses in HK-2 cells and in db/db mice. These results connect SHP2 activity to regulation of downstream ERK/NF-κB pathway status which plays an unprecedented role in renal inflammation in DN pathogenesis. However, it should be noted that ERK and NF-κB are not the sole effectors downstream SHP2 kinase, and other biological effects of SHP2 inhibition with PHPS1 on DN can not be ruled out and remain uncertain, which requires further studies.

### Supplementary Information

**Additional file 1: Fig. S1. **The parameters of mice used in this study. The blood glucose levels (A) and body weight (B) of the WT and db/db mice were measured every 2 weeks. Each group included 6 mice. One-way ANOVA test followed by a Holm-Sidak post-test, **, *p* < 0.01.

**Additional file 2:** **Fig. S2.** Correlation between SHP2 activity and inflammation degree in DN patients. Correlation analysis of integrated optical density (IOD) of p-SHP2 expression with that of CD3^+^ (A) and CCL2 (B). Pearson’s correlation analysis.

## Data Availability

The datasets generated and/or used during the present study are available from the corresponding author upon reasonable request.
