# Supplementary figures and images for: Targeting Src homology phosphatase 2 ameliorates mouse diabetic nephropathy by attenuating ERK/NF-κB pathway-mediated renal inflammation
Source: Cell Commun Signal. 2023 Dec 18;21:362. doi: 10.1186/s12964-023-01394-9 (PMC10729421; doi:10.1186/s12964-023-01394-9)

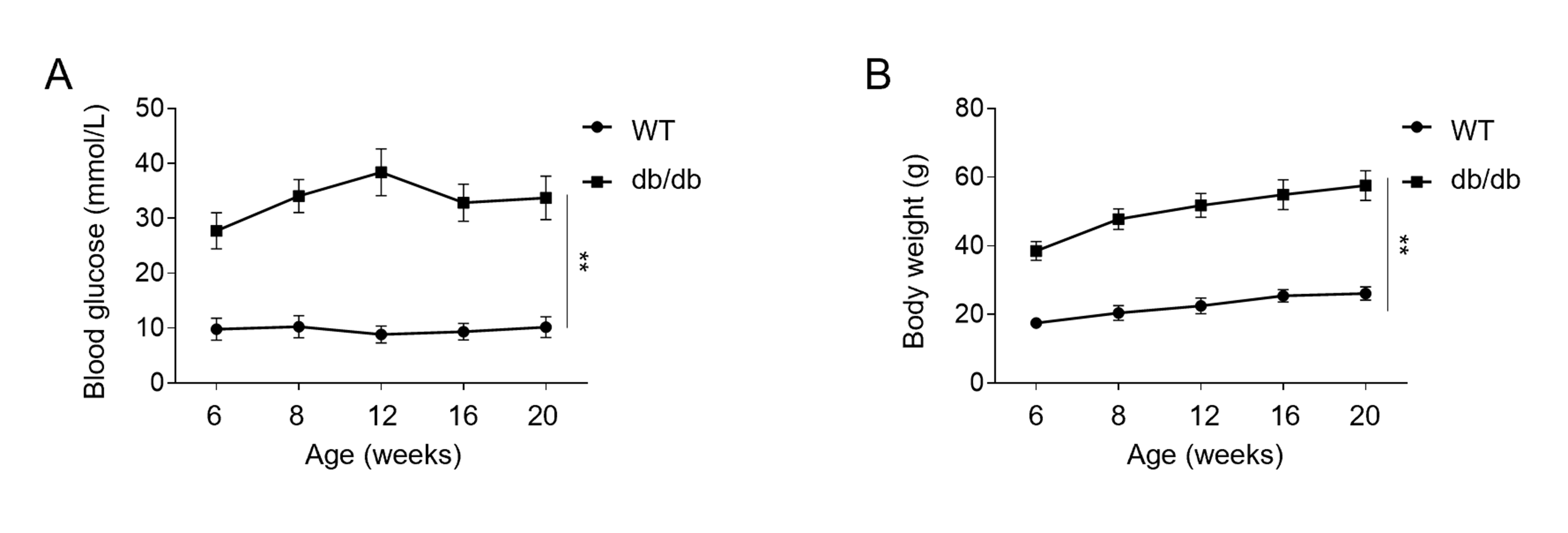

Supplement: Supplementary file 2 — Additional file 1: Fig. S1. The parameters of mice used in this study. The blood glucose levels (A) and body weight (B) of the WT and db/db mice were measured every 2 weeks. Each group included 6 mice. One-way ANOVA test followed by a Holm-Sidak post-test, **, p < 0.01. [file 12964_2023_1394_MOESM1_ESM.tif]

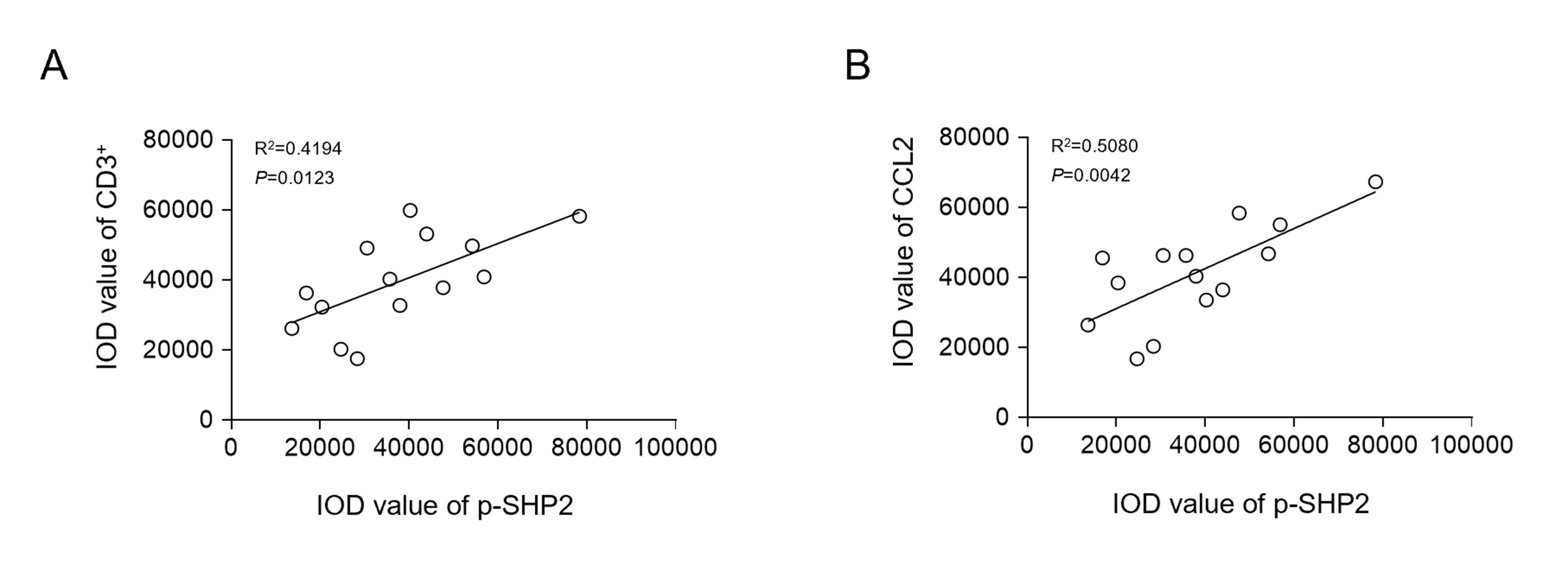

Supplement: Supplementary file 3 — Additional file 2: Fig. S2. Correlation between SHP2 activity and inflammation degree in DN patients. Correlation analysis of integrated optical density (IOD) of p-SHP2 expression with that of CD3+ (A) and CCL2 (B). Pearson’s correlation analysis. [file 12964_2023_1394_MOESM2_ESM.tif]
